# Multi-component based cross correlation beat detection in electrocardiogram analysis

**DOI:** 10.1186/1475-925X-3-26

**Published:** 2004-07-23

**Authors:** Thorsten Last, Chris D Nugent, Frank J Owens

**Affiliations:** 1 School of Electrical and Mechanical Engineering, Faculty of Engineering, University of Ulster at Jordanstown, Northern Ireland; 2 School of Computing and Mathematics, Faculty of Engineering, University of Ulster at Jordanstown, Northern Ireland

## Abstract

**Background:**

The first stage in computerised processing of the electrocardiogram is beat detection. This involves identifying all cardiac cycles and locating the position of the beginning and end of each of the identifiable waveform components. The accuracy at which beat detection is performed has significant impact on the overall classification performance, hence efforts are still being made to improve this process.

**Methods:**

A new beat detection approach is proposed based on the fundamentals of cross correlation and compared with two benchmarking approaches of non-syntactic and cross correlation beat detection. The new approach can be considered to be a multi-component based variant of traditional cross correlation where each of the individual inter-wave components are sought in isolation as opposed to being sought in one complete process. Each of three techniques were compared based on their performance in detecting the P wave, QRS complex and T wave in addition to onset and offset markers for 3000 cardiac cycles.

**Results:**

Results indicated that the approach of multi-component based cross correlation exceeded the performance of the two benchmarking techniques by firstly correctly detecting more cardiac cycles and secondly provided the most accurate marker insertion in 7 out of the 8 categories tested.

**Conclusion:**

The main benefit of the multi-component based cross correlation algorithm is seen to be firstly its ability to successfully detect cardiac cycles and secondly the accurate insertion of the beat markers based on pre-defined values as opposed to performing individual gradient searches for wave onsets and offsets following fiducial point location.

## Background

Computerised classification of the electrocardiogram (ECG) is a complex and multi staged process. The overall goal is to determine if the patient is 'normal' and may remain untreated, or whether the patient exhibits any cardiac abnormalities requiring treatment. The classification of the ECG by computerised techniques has been an active area of research for more than 4 decades. A plethora of algorithmic techniques have been applied and developed [1,2] all with the common goal of enhancing the classification accuracy and becoming as reliable and successful as expert cardiologists. The process can be divided into a number of sequential stages. Pre-processing stages of Beat Detection and Feature Extraction/Selection provide suitable information from the recorded ECG in the form of a digitised feature vector [3]. This can be considered to describe the current morphology of the recorded signal, hence, following processing by the classification algorithm [1,2], a set of suggestive diagnostic statements can be produced.

**Figure 1:**
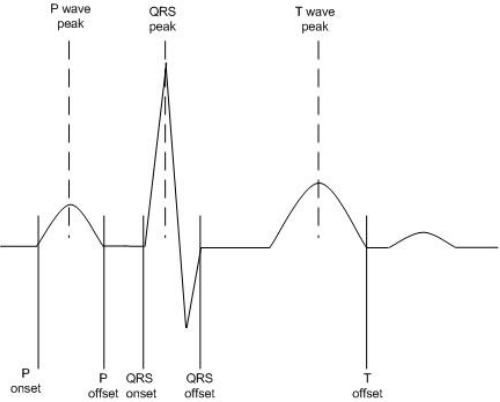
P wave, QRS complex and T wave beat markers inserted into ECG recording.

With a multi-stage computerised approach, the overall classification capabilities of the system are highly dependent on the early stages of processing, i.e., accurate detection of each ECG complex in all recorded leads. Hence the necessity of a reliable beat detection algorithm is of paramount importance. Regardless of the approach employed to analyse and classify the ECG signal, all require accurate detection of each QRS complex. Beat detection algorithms are designed with two main objectives. Firstly, the algorithm employed should provide reliable detection of each cardiac cycle. Secondly, the temporal location of the reference points should be described accurately. In general terms detection of each cardiac cycle involves the location of a fiducial point, usually taken as the peak amplitude of the R-wave or of the QRS complex. From this it is then possible to detect markers for the other interwave components (if present) and features of the ECG; QRS onset and offset, P onset and offset and T offset (Figure 1).

Two of the most commonly employed approaches to beat detection involve the use of established non-syntactic algorithms [3-5] or cross correlation (CC) algorithms [3,6]. A brief overview of each approach follows.

### Non-syntactic beat detection

A non-syntactic approach to beat detection normally involves an algorithm based on two distinct steps; a pre-processor and a decision rule. The function of the pre-processing stage is to enhance the QRS complex and suppress all other components of the recorded signal i.e. P and T waves and noise artefacts. This is achieved through firstly applying a linear filter to extract the required frequencies, followed by a non-linear transformation with the goals of providing a single positive peak for each QRS complex. The output from the non-linear transformation is then processed by a thresholding function to indicate the presence or absence of a QRS complex. Following location of a reference point remaining inter-wave components can be located to the left (P wave) and the right (T wave) of the QRS complex.

**Figure 2:**
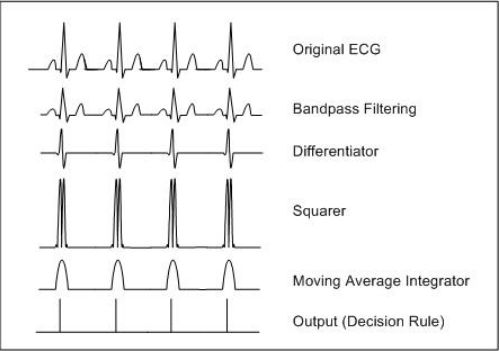
Non-syntactic non-linear transformations transforming original ECG signal into a series of impulse like outputs.

A number of non-linear transformations exist which can be applied and can occur in a number of different permutations. A commonly adopted approach is conceptualised in Figure 2. The primary stage of bandpass filtering (approximately 5–25 Hz) reduces the noise artefacts that may be present in the signal. Detection of the fiducial point need not necessarily occur on the original signal, but can be considered advantageous once some signal transformations have been applied, hence the inclusion of the differentiator, squaring function and moving window integrator. The differentiation stage acts as a highpass filter, exploiting the slope characteristics of the QRS complex. The squaring function is also favourable to the high-frequency components of the signal and serves the purpose for signal rectification. The integrator includes information relating to the duration of the QRS complex, which is recognised in electrocardiography as normally having the longest duration of any component in the ECG. QRS complexes cannot occur physiologically closer than 200 ms in succession. Therefore it is common practice once a QRS complex is detected, that a 200 ms refractory blanking period is initiated. This eliminates the condition where the same QRS complex is detected twice or even a T wave is mistaken for a QRS complex. (For a more detailed review of studies applying non-syntactic beat detection see [3].)

### Cross Correlation based beat detection

The CC function can be used to measure the similarities between two signals [7]. This process entails the computation of the sum of the products of corresponding pairs of points of two signals, within a specified time frame, or window. The process also takes into consideration any potential phase differences between the two signals via the inclusion of a lead or lag term. The formula for CC is represented as:



where *N *represents the number of samples, *j *is the lag factor and *x*_1 _and *x*_2 _are the two signals. To normalise the results based on the number of sample points, the factor 1/*N *is introduced. When the value of *r*_(12) _is maximal, this is considered as the point of maximal similarity between the two waveforms. As the required amount of lagging between the two signals is initially unknown, various degrees of lags within the specified correlation interval must be performed.

It is possible to use CC for the purposes of beat detection, by locating the point of maximal similarity between an ECG signal and a predefined template and hence identifying the temporal location of the QRS complex. It is also necessary for the CC algorithm to store a template or a reference signal of the ECG signal. The origin of the template may be from a variety of sources. It may be of an adaptive nature, whereby a section of the patient's recorded ECG is averaged and stored prior to the analysis. Alternatively, a mathematical model may be used or a collection of ECG recordings from a database used to produce a generic template.

Many studies in the past have successfully reported the use of CC as a means of automated beat detection. Abboud *et al*. [6] used the CC function, calculated using the cross spectrum and fast Fourier transform algorithm for extrastoyle rejection and location of fiducial points. Variations to the CC algorithm have also been successfully reported. These were considered to be more computationally efficient as they do not require the intense multiplicative processes associated with CC, but may be based on for example weighted correlation of differences methods [8] and average magnitude cross difference methods [9]. Methods of CC have generally focused on the usage of one waveform as the basis for the template, for example the QRS complex. The algorithms of Abboud *et al*. [6] were adapted by Govrin *et al*. [10] to facilitate the location via CC of both the P waves and QRS complexes. Templates of both waves were used with unknown ECG traces to identify the individual components of each cardiac cycle.

It is the aim of the current study to investigate the potential of adapting the general CC based approach as an accurate and reliable means for beat detection. This approach in the past has been shown to be successful and hence is investigated with the aims of further enhancement. Individual templates for P waves, QRS complexes and T waves are generated and the CC approach applied individually to identify each of these components as opposed to a one template CC based approach. Standard approaches have also been developed for benchmarking purposes.

The structure of the remainder of the paper is as follows: Section 2 describes the new beat detection method based on the fundamentals of CC. In addition a description of the benchmarking methods developed are also presented. Section 3 describes the structure of the data set and presents the results and discussions for the beat detection algorithms. Final conclusions to the study are presented in Section 4.

## Methods

The aims of the current study were to investigate the possibilities of developing a new approach to beat detection which would offer enhanced accuracy and reliability in comparison to established techniques. Two different approaches, one based on a standard non-syntactic approach and one based on a standard CC approach were developed for the purposes of benchmarking. Developments of both of these algorithms were based on existing published approaches. A new approach, based on a multi-component based CC algorithm was additionally developed. Details of the 3 beat detection approaches are as follows.

### Non-syntactic approach

The first stage of the non-syntactic approach is the inclusion of a bandpass filter, centred at 17 Hz. The purpose of this is to isolate the predominant QRS energy and attenuate the low-frequency characteristics of the P and T waves, any baseline drift present and the higher frequencies associated with electromyographic noise and power line interference [11]. (The passband that maximises the QRS energy is approximately in the 5–25 Hz range.) An FIR bandpass filter was implemented with the difference equation expressed in Equation 2:



where *y*_*i *_is the filtered output sequence, *x*_*i *_is the input ECG signal and *b*_*q *_represents the coefficients calculated with the Remez exchange design algorithm [12]. To produce the necessary stage of differentiation, a five-point derivative with the second term equal to zero (as represented in Equation 3) was employed.

*H*(*z*) = 0.1(2 + *z*^-1 ^- *z*^-3 ^+ 2*z*^-4^)     (3)

This has been chosen as the function *H*(*z*) in Equation 3 behaves in a similar manner to a parabolic smoothing filter and does not amplify any high-frequency noise. The advantage of such a filter is that it goes to zero at half of the sampling frequency. The non-linear transformation is performed by the point-by-point squaring of the signal samples. This derivative approximates an ideal derivative in the dc through to 30 Hz frequency range. This is the necessary frequency range since all higher frequencies are significantly attenuated by the bandpass filter. Finally, the squared waveform is passed through a moving window integrator. A window integrator with the difference equation as presented in Equation 4 was employed.



where *N *is the number of samples in the width of the moving window. The integrator sums the area under the squared waveform over an approximate 180 ms interval, advances one sample interval and integrates a new 180 ms window.

To locate the fiducial point, the maximum of the QRS complex is required. Following application of the aforementioned processes to the recorded signal, the maximum amplitude of the resultant signal is sought. An initial threshold value based on 80% of the maximum amplitude located during an initial training period is used to locate potential QRS complexes. Within a 125 ms window, following exceeding this value, the maximum amplitude located is considered to be the fiducial point. A refractory blanking stage of 200 ms is employed prior to repeating the process for the next cardiac cycle. The markers for the onset and offset are inserted following gradient searches to the right and left of the fiducial point within a window of 200 ms on each side. A thresholding technique was exercised, whereby following the location of 6% of the gradient, for a duration of at least 25 ms, to the left and right of the fiducial point the start and end points were identified respectively.

Prior to location of the T wave offset, the original signal was low-pass filtered at 12 Hz in an effort to maximise the frequency content of the T wave whilst suppressing the remaining inter-wave components. With knowledge of the end of the QRS-complex and the beginning of the next adjacent QRS-complex a value of the peak of the T wave, i.e. the maximum value within the identified window, was calculated. The T-offset is located based on a gradient descent thresholding search method. The condition of thresholding was set at 25% of the maximum gradient of the T wave, for a duration of at least 25 ms, to the right of its peak.

Following filtering and smoothing, the P wave was sought in a window of 300 ms prior to the QRS onset. Similar to the T wave offset location, the maximum (or peak) is located within the defined window prior to the location of the onsets and offsets. The onset is located following detection of 75% of the maximum gradient, for a duration of at least 25 ms, to the left of the peak and the offset is located following detection of 75% of the maximum gradient, for a duration of at least 25 ms, to the right of the peak.

### CC approach

The necessary templates required for the CC were generated during an initial training stage prior to any CC based analysis. During this stage templates required on an individual lead-per-lead basis were generated by averaging cardiac cycles in the initial section of the patient's ECG recording. This requirement for a training stage generally results in CC based approaches being more suitable for ambulatory processing situations, for example Holter recordings, as opposed to analysis of short term rest ECGs. Given that infinite signals are to be analysed, the CC function for a lag *k *can be defined as in Equation 5:



where the correlation coefficient is defined as:



where *z *can be set to either *x *or *y*. The CC approach is only used to detect the fiducial point. The signal is cross correlated with a complete PQRST template. The correlation function calculates a number of correlation coefficients and returns the position of the highest value. Providing this value is greater than a predefined threshold, a QRS is deemed detected. The threshold value can be changed for each signal and template, if required. This ensures adaptability to each individual patient's recording.

The QRS onset and offset as well as the P wave onset and offset and T wave offset are detected with similar rule-based gradient search techniques as described in the non-syntactic approach in the previous section. The main difference being that the search windows in which the gradient searches are performed are dependent upon the distances specified in the template with which the ECG signal is cross correlated.

### Multi-component based CC algorithm

**Figure 3:**
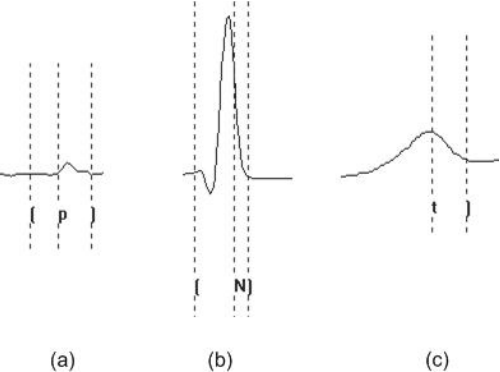
An example of templates used by the multi-component based CC approach for (a) P wave (b) QRS complex (c) T wave.

With the proposed multi-component based CC detection method, separate templates for each interwave component are used. Each template consists of an individual component of the complete cardiac cycle, detailing accurate marker positions for the respective inter-wave component onset and offset. In the given approach, 3 templates were employed, one for the P wave, one for the QRS complex and one for the T wave (Figure 3).

**Figure 4:**
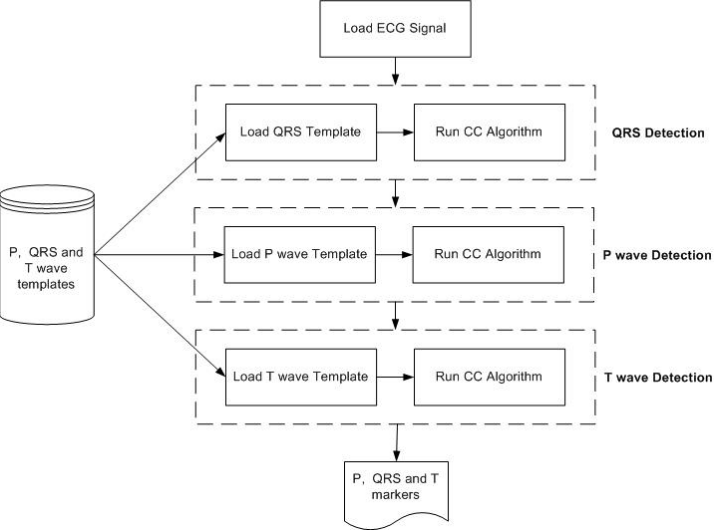
Basic structure of the multi-component based CC detection method using 3 separate templates.

The detection method is based purely on CC and is structured into a number of procedural steps as shown in Figure 4. The CC function employed in all steps is as described in Equation 5. The only difference is that for each interwave component sought, differing templates are used within the algorithm. The first step of the process is concerned with the location of the QRS complex. With this process, a QRS template is cross correlated with the ECG signal. The next step is the detection of the P wave, which is performed using the same CC function, but in this case, the template is representative of the patient's P wave. The last stage involves the detection of the T wave. In this process, a template, representative of the patient's T wave is cross correlated with the ECG signal using the same CC function as with the previous 2 steps.

In each case the value indicated as being the point of maximum similarity, i.e. the highest correlation coefficient, between the ECG and the template, within the given correlation interval is compared with a pre-defined threshold. The threshold level, used to initially locate the peak amplitude can be varied in the algorithm if required and is established during a pre-learning phase. If the amplitude of the value exceeds the threshold, a waveform detection is considered as having occurred, otherwise the process is repeated with a new portion of the signal. Markers for the QRS onset, QRS offset, P wave onset and P wave offset, as well as the T wave offset form part of the templates used during the CC process. Hence as soon as the individual interwave components are detected, the markers are generated automatically based on the templates used with no requirement to perform any means of gradient based searching.

## Results and Discussion

To examine the efficiency of the algorithms, excerpts from the already established QT database were employed [13]. This database has been designed specifically for the evaluation of algorithms which detect waveform boundaries in the ECG. The database has approximately 100 records, each record consisting of 15 minute excerpts of two-channel digitised ECGs. The recordings were chosen to include a broad variety of QRS and ST-T morphologies. The records within the QT database were chosen from the MIT-BIH database, the European ST-T database and several other ECG databases collected at Boston's Beth Israel Deaconess Medical Centre. For each record, a minimum of 30 beats have been manually annotated by clinical experts. For each annotated beat, the following markers have been inserted; P wave onset, P wave offset and P wave peak amplitude, QRS onset, QRS offset and QRS peak amplitude and T wave offset and T wave peak amplitude. For the purposes of the given study, each algorithm was exposed to 3000 beats from this database.

### Comparison of beat detection algorithms

To quantify the accuracy of each of the three aforementioned beat detection algorithms in terms of their correct positioning of beat markers, measurements of mean error (*me*) and standard deviation (*SD*) of this error were used. The *me *value is used to determine how close the detector is to the annotated markers, with the *SD *value providing information relating to the stability of the detection criteria. For the purposes of validation, the database used for testing [13] has associated with it a set of tolerance values for each of the beat markers. These measures can be considered to be the minimum values that should be expected with any automatic algorithm. The accuracy with which the automated algorithms performed the detection was compared with manually annotated and clinical validated beat markers. Such comparisons with a gold standard and subsequently with other automated approaches operating on the same data sets adhere to recommended approaches of comparison of automated medical decision support systems [14].

Average values for the *SD *(Equation 6) of the *me *can be generated with the following equation:



Where *x*_*id *_is the detected marker position for ECG trace *i *as identified by the algorithms used and *x*_*im *_is the original stored marker position from the database annotated by experts for ECG trace *i*.

Table 1 shows the performance of each of the algorithms in detecting the 3000 cardiac cycles from the test set. Table 2 indicates the performance of each of the algorithms in comparison with the accepted tolerances for marker insertion.

**Table 1:** Results of performance following exposure to 3000 cardiac cycles for each algorithm.

	**Non-Syntactic**	**CC**	**Multi-component based CC**
Number of QRS detected out of possible 3000	2850	2799	2931

**Table 2:** Results of marker accuracy following exposure to 3000 cardiac cycles for each algorithm.

**Marker**	**Tolerance **SD ms	**Non-Syntactic **SD ms	**CC **SD ms	**Multi-component based CC **SD ms
P-onset	**10.2**	22.6	22.1	11.9
P amplitude		23.4	23.8	7.8
P-offset	**12.7**	16.7	19.7	11.6
QRS-onset	**6.5**	10.4	10.1	6.6
QRS amplitude		14.3	1.8	1.8
QRS-offset	**11.6**	12.8	13.1	6.9
T amplitude		19.2	21.4	8.2
T-offset	**30.6**	18.7	20.6	14.6

In terms of the overall accuracy in detecting cardiac cycles, as shown in Table 1, the multi-component based CC approach provided the best results. The advantages of considering only the QRS complex during the CC process offers an improvement in detection of each cardiac cycle. It can be considered that the PQRST wave poses a more complex situation to achieve an accurate measurement of maximum similarity during the correlation process than with a template which only represents the QRS portion of the ECG.

Considering the accuracy with which the three algorithms were able to detect the correct position for the marker insertion as shown in Table 2 indicates that the multi-component based CC approach outperformed the two benchmarking algorithms in 7 out of the 8 marker insertion positions. In the remaining case (QRS amplitude) the multi-component based CC achieved a similar *SD *of 1.8 ms with the traditional CC approach. The non-syntactic and CC approaches performed to a similar level with the former providing superior results in 5 out of the 8 marker values. The *SD *values, however, only differ slightly. This can be considered to be the result of both techniques using a similar approach of gradient descent searching to identify onsets and offsets. The difference in the results can be attributed to the different manner in which each technique defines the search window within which the gradient searching is performed.

The increased accuracy of marker insertion of the multi-component based CC approach can be attributed to a number of factors. The major factor being the avoidance of any gradient searching techniques for the marker positioning as is required by the two benchmarking approaches. As the multi-component based CC approach has pre-defined marker positions in-built as an inherent feature of its design, the ability to detect the fiducial point accurately is the most important process the algorithm initially undertakes. Following this process, markers are inserted based on the values stored within the templates used during the correlation process. Methods used in the two benchmarking techniques of gradient searches are prone to false detection of local gradients and noise still remaining in the signal under examination.

Considering the accepted tolerances for the 5 markers as identified in Table 2, the multi-component based CC approach conformed to 4 of these (QRS onset marginally higher – 6.6 ms vs 6.5 ms) with the P onset lying outside of what was considered to be an acceptable range. Given the historical difficulty of P wave detection and accurate marker location reported by many studies [15], this is not initially considered to be a drawback of the algorithm, but more an indication of an area requiring further improvement of the approach.

Overall the multi-component based CC approach outperformed the two benchmarking techniques in both accuracy of cardiac detection and marker insertions, however, 2 parameters within the algorithm were found to have a significant influence on the algorithm's performance; correlation interval and threshold parameter. Values for these parameters can be established during a training period.

### Correlation interval

**Figure 5:**
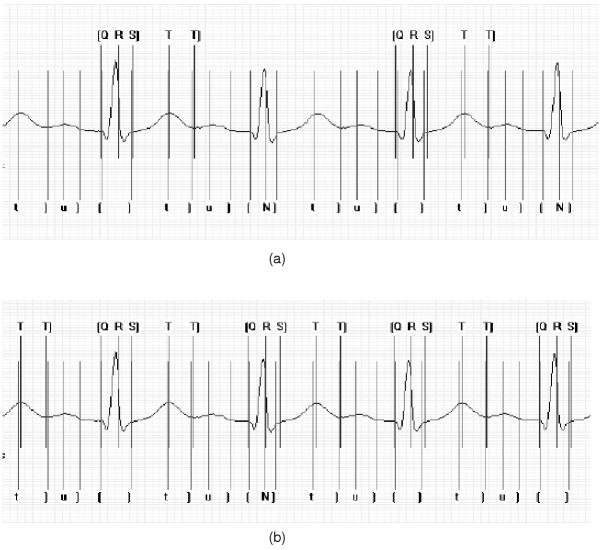
Insertion of beat markers with a correlation interval of (a) 560 ms (b) 280 ms. Markers included on the bottom of the trace are those indicated and inserted by clinical experts. The notation used is as follows: t] represents the position of the markers for the t wave amplitude and t wave offset respectively; [N] represents the position of the markers for the QRS onset, peak and offset respectively, where N is used to represent a QRS complex; u] represents the position of the markers for the u wave amplitude and u wave offset respectively. Markers included on the top of the trace are those indicated following automated processing. The notation used is as follows: [Q R S] represents the position of the markers for the QRS onset, QRS peak and QRS offset respectively; T T] represents the position of the markers for the T wave peak and T wave offset respectively. In instances where no markers have been indicated, the algorithm has failed to correctly detect the waveform boundaries and peaks.

Results following the testing process indicated that care must be taken when selecting the appropriate correlation interval for CC based approaches. If this value is too large, interwave components of adjacent waves maybe considered during the correlation process. On the other hand, if the interval is too small, the templates used during the correlation process may not have the ability to discriminate between different desired portions of the underlying signal. Figure 5 shows the multi-component based CC approach processing an excerpt from one record with a correlation interval of 560 ms and 280 ms. The markers indicated at the top of the trace are those automatically inserted by the algorithm. The markers indicated at the bottom of the trace are those which have been inserted manually by clinical experts.

In the first instance detection rates are low as the interval is too large, however, when the interval is reduced to 280 ms, the rate of detection of the markers for the algorithm increases significantly. Given that under normal conditions the duration of the QRS complex is in the region of 100 ms [16] and a second QRS complex cannot physiologically occur for a further 200 ms, this would suggest a value of the correlation interval in the region of 300 ms as a suitable choice.

### Threshold parameter

**Figure 6:**
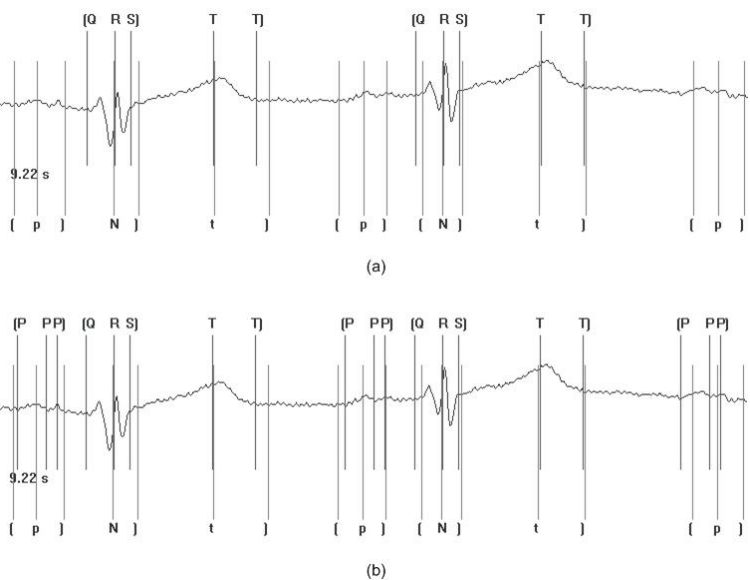
Insertion of beat markers for threshold values of (a) 85% (b) 60% of training averages. Markers included on the bottom of the trace are those indicated and inserted by clinical experts. The notation used is as follows: t] represents the position of the markers for the t wave amplitude and t wave offset respectively; [N] represents the position of the markers for the QRS onset, peak and offset respectively, where N is used to represent a QRS complex; [p] represents the position of the markers for the p wave onset, amplitude and p wave offset respectively. Markers included on the top of the trace are those indicated following automated processing. The notation used is as follows: [Q R S] represents the position of the markers for the QRS onset, QRS peak and QRS offset respectively; T T] represents the position of the markers for the T wave peak and T wave offset respectively; [P P P] represents the position of the markers for the p wave onset, peak and offset respectively. In instances where no markers have been indicated, the algorithm has failed to correctly detect the waveform boundaries and peaks.

The threshold parameter can be considered to be the minimum value for a correlation result to be considered as a true wave detection. For example, P waves can be considered to have relatively low energy content. If the threshold value is initially set at too large a value then it will not be possible for the point at which the CC function returns the point of maximum similarity to exceed this and subsequently indicate a waveform detection. Figure 6 shows the multi-component based CC algorithm with threshold values of 85% and 60% of training waveform peak amplitude averages. As can be seen it was found necessary that the final thresholding check following the CC considered a lower percentage of the average signal amplitude to ensure successful detection of the wave.

The multi-component based CC approach is affected by both the correlation interval and threshold parameters of the algorithm, hence a training/tuning process is required. Each of the other two algorithms suffer from similar inherent algorithmic drawbacks and hence this is not considered to be a disadvantage of the approach provided it is taken into consideration during application of the algorithm.

### Abnormal recordings

Although, as previously mentioned, the QT database has a large variety of abnormal ECG recordings, two specific examples are highlighted at this point to further compare the performance of the multi-component based CC approach and the non-syntactic based approach. In the first instance a recording where an inverted T wave is present is examined and in the second instance a recording similar to conditions exhibited by First Degree Heart Block is examined.

**Figure 7:**
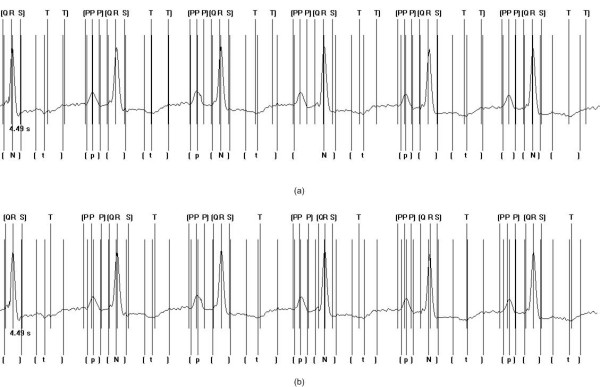
Application of the multi-component based CC approach (a) and the non-syntactic based approach (b) to abnormal ECG waveforms. In this case, an inverted T wave is present. Markers included on the bottom of the trace are those indicated and inserted by clinical experts. The notation used is as follows: t] represents the position of the markers for the t wave amplitude and t wave offset respectively; [N] represents the position of the markers for the QRS onset, peak and offset respectively, where N is used to represent a QRS complex; [p] represents the position of the markers for the p wave onset, amplitude and p wave offset respectively. Markers included on the top of the trace are those indicated following automated processing. The notation used is as follows: [Q R S] represents the position of the markers for the QRS onset, QRS peak and QRS offset respectively; T T] represents the position of the markers for the T wave peak and T wave offset respectively; [P P P] represents the position of the markers for the p wave onset, peak and offset respectively. In instances where no markers have been indicated, the algorithm has failed to correctly detect the waveform boundaries and peaks.

Figure 7(a) shows the correct insertion of the markers for the multi-component based CC approach and Figure 7(b) shows the insertion of the markers with the non-syntactic based approach. As can be seen, comparing these two techniques, both have the ability to correctly detect the peak of the T wave, however, the multi-component based CC approach has the ability to detect in all instances the end of the T wave, which was not correctly detected in any of the cases analysed by the non-syntactic approach. The ability to know the shape and form of each component of the ECG waveform in advance has shown here the further benefits of the multi-component based CC approach and in addition showed its ability in instances of non-normal ECG recordings to perform successfully.

**Figure 8:**
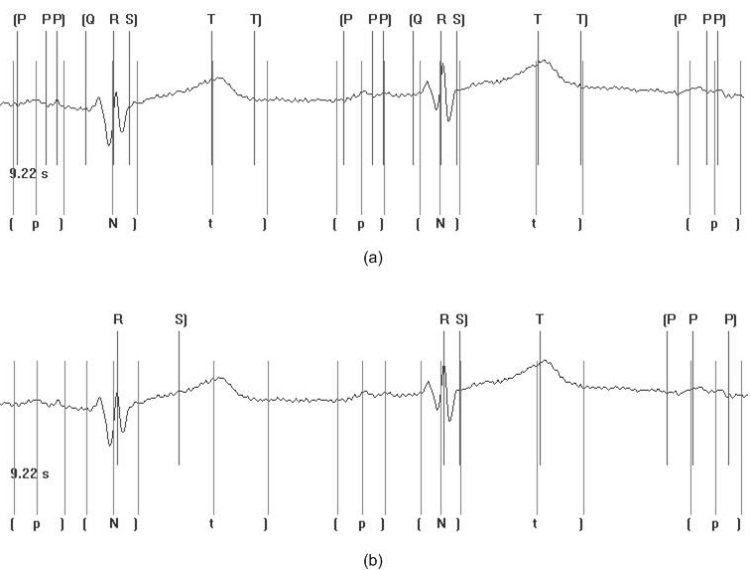
Application of the multi-component based CC approach (a) and the non-syntactic based approach (b) to abnormal ECG waveform. In this case First Degree Heart Block is present in the recording. Markers included on the bottom of the trace are those indicated and inserted by clinical experts. The notation used is as follows: t] represents the position of the markers for the t wave amplitude and t wave offset respectively; [N] represents the position of the markers for the QRS onset, peak and offset respectively, where N is used to represent a QRS complex; [p] represents the position of the markers for the p wave onset, amplitude and p wave offset respectively. Markers included on the top of the trace are those indicated following automated processing. The notation used is as follows: [Q R S] represents the position of the markers for the QRS onset, QRS peak and QRS offset respectively; T T] represents the position of the markers for the T wave peak and T wave offset respectively; [P P P] represents the position of the markers for the p wave onset, peak and offset respectively. In instances where no markers have been indicated, the algorithm has failed to correctly detect the waveform boundaries and peaks.

Figure 8(a) and 8(b) show the results of the multi-component based CC approach and the non-syntactic based approach respectively given the instance of First Degree Heart Block. As can be seen the multi-component based CC approach outperforms the non-syntactic based approach, with the ability to detect all elements of the QRS complex. The non-syntactic based approach has only the ability to detect the peak of the QRS complex. The complexity associated with the abnormality has deemed the algorithm unable to analyse any further interwave components. The benefits of matching the waveform with a template previously established for the person under examination offers the ability to specifically tailor to the recordings under investigation as opposed to generically processing the signal as with the non-syntactic approach.

## Conclusions

The accurate detection of the interwave components of the ECG can be considered to significantly effect the overall performance of the computerised classification process. Three approaches to beat detection were developed and extensively tested on 3000 cardiac cycles to assess their performance. Non-syntactic beat detection and CC algorithms were used as means of benchmarking algorithms. A new approach of multi-component based CC was proposed and results showed it to out perform the two benchmarking techniques in both accuracy of cardiac cycle detection and marker insertions. The multi-component approach identified beat markers based on 3 individual CC processes addressing the QRS complex, the P wave and the T wave individually. For each of these correlation steps, once a correlation match had been established, markers were inserted for onsets and offsets based on predefined values. It is proposed that the increase in performance of this approach in comparison with the benchmarks can be attributed to the lack of heuristic gradient searching required for marker insertion. In addition, the ability to match a portion of the ECG signal, namely the QRS, during CC as opposed to matching to the entire PQRST reduces the complexity of the overall process and subsequently enhances performance.

Overall, the results have shown the benefits of employing a multi-component based CC approach. Further studies are currently underway to investigate the performance of the algorithms under instances of noise conditions and further variants of non-normal ECG recordings and in addition possible improvements to the stage of P wave detection.

## Authors' contributions

TL carried out the development of the algorithms and performed the testing procedures. TL, CDN and FJO collaboratively designed the study. TL, CDN and FJO co-authored the paper.
